# Evaluation of cutaneous sensory block area following a novel approach to transversus abdominis plane block: an observational study

**DOI:** 10.3389/fmed.2025.1605364

**Published:** 2025-09-12

**Authors:** Cheng Xu, Sai Kong, Yongzhu Chen, Jie Lu, Aizhong Wang

**Affiliations:** Department of Anaesthesiology, Shanghai Sixth People's Hospital Affiliated to Shanghai Jiao Tong University School of Medicine, Shanghai, China

**Keywords:** transversus abdominis plane block, cutaneous sensory block area, regional anesthesia, multimodal analgesia, laparoscopic cholecystectomy, ultrasound-guided

## Abstract

**Background:**

Ultrasound-guided transversus abdominis plane (US-TAP) block is currently used as part of a multimodal analgesic regimen in anterior abdominal wall surgery, but the distribution of cutaneous sensory block area (CSBA) shows significant interindividual variation. We predeveloped a novel US-TAP block approach, and this study aims to assess the CSBA following the novel US-TAP block approach.

**Methods:**

Sixteen patients undergoing elective laparoscopic cholecystectomy (LC) received bilateral novel US-TAP blocks with a total of 40 mL of 2.5 mg/mL ropivacaine. Measurements were taken 45 min after block administration. CSBA was mapped using cold sensation and a sterile marker, photodocumented, and transferred to a transparency. The area of the CSBA was calculated from the transparencies.

**Results:**

The median CSBA of the novel US-TAP approach was 332 cm^2^ (IQR 297–413 cm^2^; range 258–466 cm^2^). In all patients, the CSBA showed wide periumbilical distribution. In all 32 unilateral blocks (100%), both epigastric and infraumbilical components were present; and in 16 of the 32 blocks (50%), the CSBA extended to the abdominal wall lateral to the vertical reference line through the anterior superior iliac spine. Fourteen patients (88%) had resting NRS scores of 3 or lower within 24 h postoperatively.

**Conclusion:**

The novel US-TAP approach produces a broad dermatomal CSBA, covering much of the abdominal wall around the umbilicus.

**Clinical trial registration:**

https://www.chictr.org.cn/showproj.html?proj=211485, ChiCTR2300077899.

## 1 Introduction

The transversus abdominis plane (TAP) block is a widely used technique for providing truncal analgesia in abdominal surgeries (1, 2). Despite its popularity, the conventional TAP block often results in inconsistent analgesia due to variable spread of the local anesthetic within the transversus abdominis plane (3, 4). Various approaches—including subcostal (5), lateral (2), and posterior (6)—have been explored to enhance the distribution of the anesthetic agent and improve analgesic outcomes. However, these modifications have not fully resolved the issue of inconsistent sensory blockade.

Comprehending block characteristics—target structures, duration, and variability—is essential for optimizing the clinical efficacy of this technique. TAP blocks theoretically target the 7th to 11th intercostal nerves, as well as the subcostal, iliohypogastric, and ilioinguinal nerves (7). The sensory effects of TAP blocks have been reported to span dermatomes from T6 to L2, depending on the specific approach (7). However, current TAP block approaches exhibit non-dermatomal distribution of the cutaneous sensory block area (CSBA), with considerable interindividual variability in CSBA size, location, and block duration (both sensory and motor) (8–10).

Although we previously described a novel TAP block approach (11), CSBA mapping has not yet been documented. This study explores the novel US-TAP approach. We hypothesized that the novel US-TAP approach would yield a broad dermatomal sensory block with more consistent characteristics. This study aimed to assess the CSBA of the novel US-TAP approach by measuring cold sensation.

## 2 Methods

### 2.1 Approvals and monitoring

This study was conducted at a single clinical site in Shanghai, China, as part of a randomized, double-blind clinical trial (ChiCTR2300077899). The Shanghai Sixth People’s Hospital Ethics Committee approved the study protocol (no. 2023-109-(1)). We collected prospective data from 16 patients who underwent elective three-port laparoscopic cholecystectomy (LC) at Shanghai Sixth People’s Hospital, a tertiary academic center affiliated with Shanghai Jiaotong University, between November 25, 2023, and April 15, 2024. All participants provided written informed consent. Reporting conforms to the STROBE (Strengthening the Reporting of Observational Studies in Epidemiology) guidelines (Supplementary material).

### 2.2 Inclusion and exclusion criteria

In this study, adult patients aged 18 to 75 years with an ASA physical status of 1 or 2, scheduled for elective three-port LC, were eligible for enrollment and recruited by research assistants during preoperative anesthesia visits. The exclusion criteria included prior abdominal wall surgery, ropivacaine allergy, coagulation disorders, chronic opioid use, significant cardiac, hepatic, renal dysfunction, psychiatric conditions, infection at the puncture site, or coagulation abnormalities.

### 2.3 Intervention

No preoperative premedication was administered in the ward. Upon entering the operating room, an 18-gauge intravenous cannula was placed in a peripheral vein of the non-operative arm, and lactated Ringer’s solution infusion was initiated. Continuous monitoring included three-lead electrocardiogram, heart rate, invasive blood pressure, respiratory rate, and pulse oximetry.

**Figure 1:**
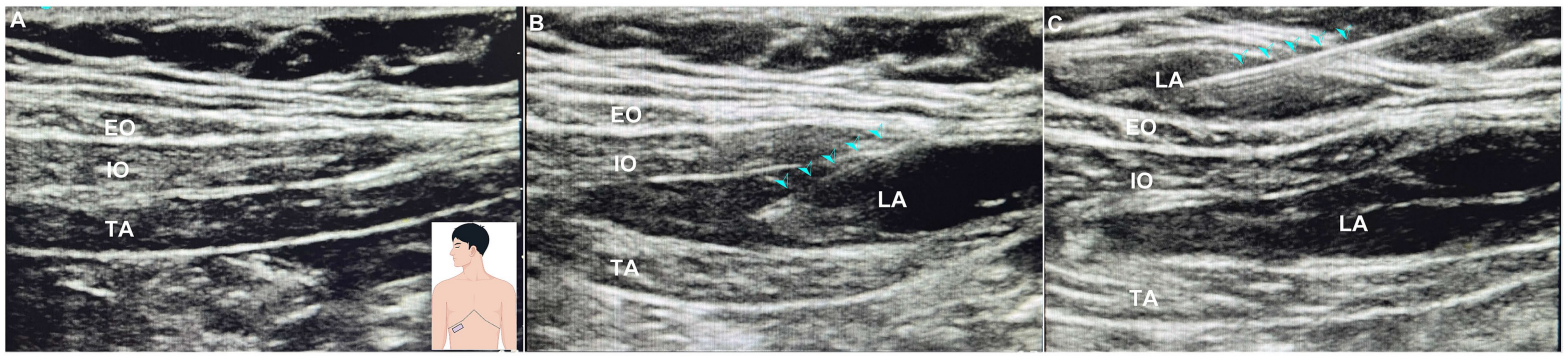
The novel ultrasound-guided transversus abdominis plane block approach. **(A)** Pre-block ultrasound probe placement and ultrasound images; **(B)** the initial injection: needle-tip placement at the fascia between the internal oblique and transversus abdominis as visualized using ultrasonographic imaging; **(C)** the second injection: needle-tip placement at the superficial fascia of the external oblique muscle as visualized using ultrasonographic imaging.

Prior to general anesthesia, while the patient was supine, an anesthesiologist skilled in the procedure administered the novel TAP block. All procedures in this trial were performed by two senior anesthesiologists (X.C. and L.J.) with extensive experience in TAP blocks. The novel TAP block technique employed a “one-stitch” approach, eliminating the need for a second needle insertion. The detailed method description has been previously published (11). Briefly, a high-frequency probe (5–13 MHz) is positioned beneath the subcostal margin and moved from medial to lateral along the subcostal region to visualize the three muscle layers: external oblique (EO), internal oblique (IO), and transversus abdominis (TA) (Figure 1A). The initial injection is directed at the fascia between the internal oblique and transversus abdominis, administering 20 mL of 0.25% ropivacaine with an 80–100 mm, 21-gauge needle (Figure 1B). The needle is then withdrawn subcutaneously, and a second injection of 20 mL of 0.25% ropivacaine is administered, targeting the superficial fascia of the external oblique muscle (Figure 1C). Ultrasonography confirmed the separation of fascial planes. No supplementary local anesthetic (LA) infiltration was administered during the perioperative period.

### 2.4 Anesthesia protocols and postoperative analgesia

Anesthesia was induced with 2 mg/kg of propofol, 0.4 μg/kg of sufentanil, and followed by 0.6 mg/kg of rocuronium for muscle relaxation. Maintenance was achieved using 2% sevoflurane in a 50% O₂ (2 L/min) and 60% air mixture. The remifentanil infusion rate was titrated between 0.05 and 0.4 mg/kg/min to maintain heart rate and blood pressure within 20% of baseline values. After surgery, neuromuscular blockade was reversed, and tracheal extubation was performed once sufficient muscle strength was regained. All patients were monitored for 60 min in the post-anesthesia care unit.

The standard analgesia protocol included 1 g of intravenous paracetamol every 8 h, 50 mg of intravenous flurbiprofen axetil every 12 h, and 1 mg/kg of tramadol; the initial doses were administered 15 min before the conclusion of surgery. Pain intensity at rest and during movement was assessed using the 0–10 Numerical Rating Scale (NRS). Intravenous flurbiprofen axetil (50 mg) was administered as a rescue analgesic if the NRS was ≥4.

### 2.5 Outcome measures

**Figure 2:**
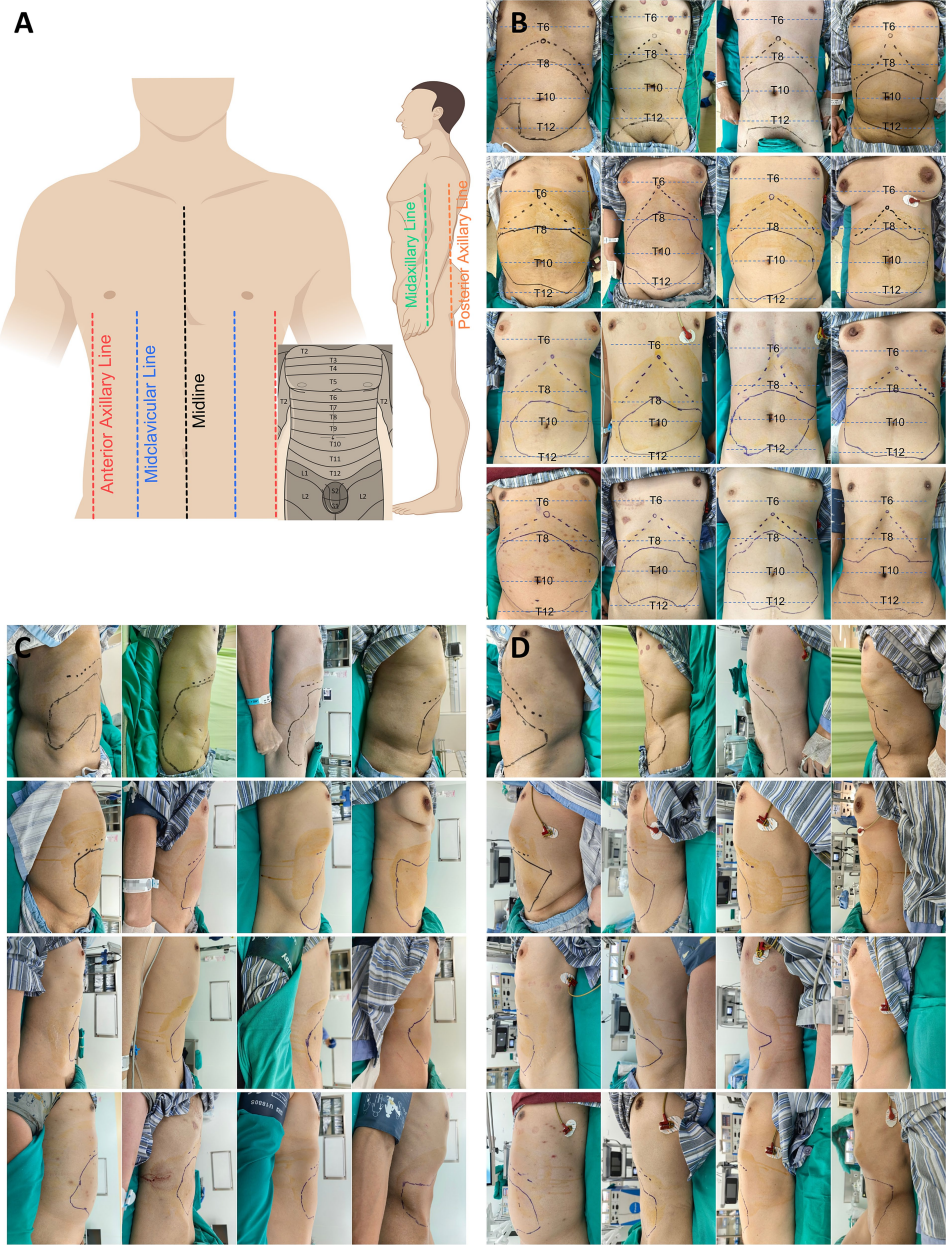
Visualisation of the cutaneous sensory block area (CSBA) after bilateral novel ultrasound-guided transversus abdominis plane block approach in 16 subjects. **(A)** Outline the skin areas of the anterior abdominal wall and anterolateral abdominal wall with vertical lines of different colors. **(B)** Frontal view. The solid line portion represents the CSBA, the dashed line represents the costal margin, and the circle represents the sternal angle. **(C)** Right lateral view; **(D)** left lateral view.

The primary outcome, the CSBA, was evaluated 45 min following block completion. CSBA was identified by applying alcohol-dipped gauze to the skin, followed by marking. A radial application technique, centered at the umbilicus, was used to tap the alcohol-soaked gauze on the abdominal wall along the midsagittal line to assess changes in cold sensation. A single investigator conducted all CSBA mapping. Patients were instructed to differentiate between an immediate cold sensation and feelings of heat or numbness. If uncertainty arose, the area was reassessed prior to concluding the examination. After confirming changes three times, markings were placed on the skin, and a connecting line was drawn to delineate the CSBA. The marked area was transferred onto transparencies. Using photographs of the transparencies, the total area and the medial, lateral, cephalad, and caudad distributions were measured with SketchAndCalc software. We also defined longitudinal surface landmarks to evaluate the likelihood of CSBA blockade from T6 to L1. The anterior abdominal region encompassed the area between the midline and both midclavicular lines. The anterolateral abdominal region extended from each midclavicular line to the corresponding midaxillary line. As illustrated in Figure 2A, a blockade was considered effective when the CSBA-affected area overlapped partially or completely with the boundary of a designated zone.

Secondary outcomes included numeric rating scale (NRS) scores at 1, 4, 12, 18, and 24 h postoperatively, along with block-related complications. In the NRS, a score of 0 indicates “no pain,” while 10 represents “the worst possible pain”.

### 2.6 Statistical analysis

Sample size estimation was deemed unnecessary due to the observational design of the study. Continuous variables are reported as median, range, and interquartile range (IQR), while categorical variables are presented as numbers with percentages. Statistical analyses were conducted using IBM SPSS Statistics version 26 (IBM) and Rex version 3.5.3 (RexSoft Inc.).

## 3 Results

### 3.1 Study participants

**Figure 3:**
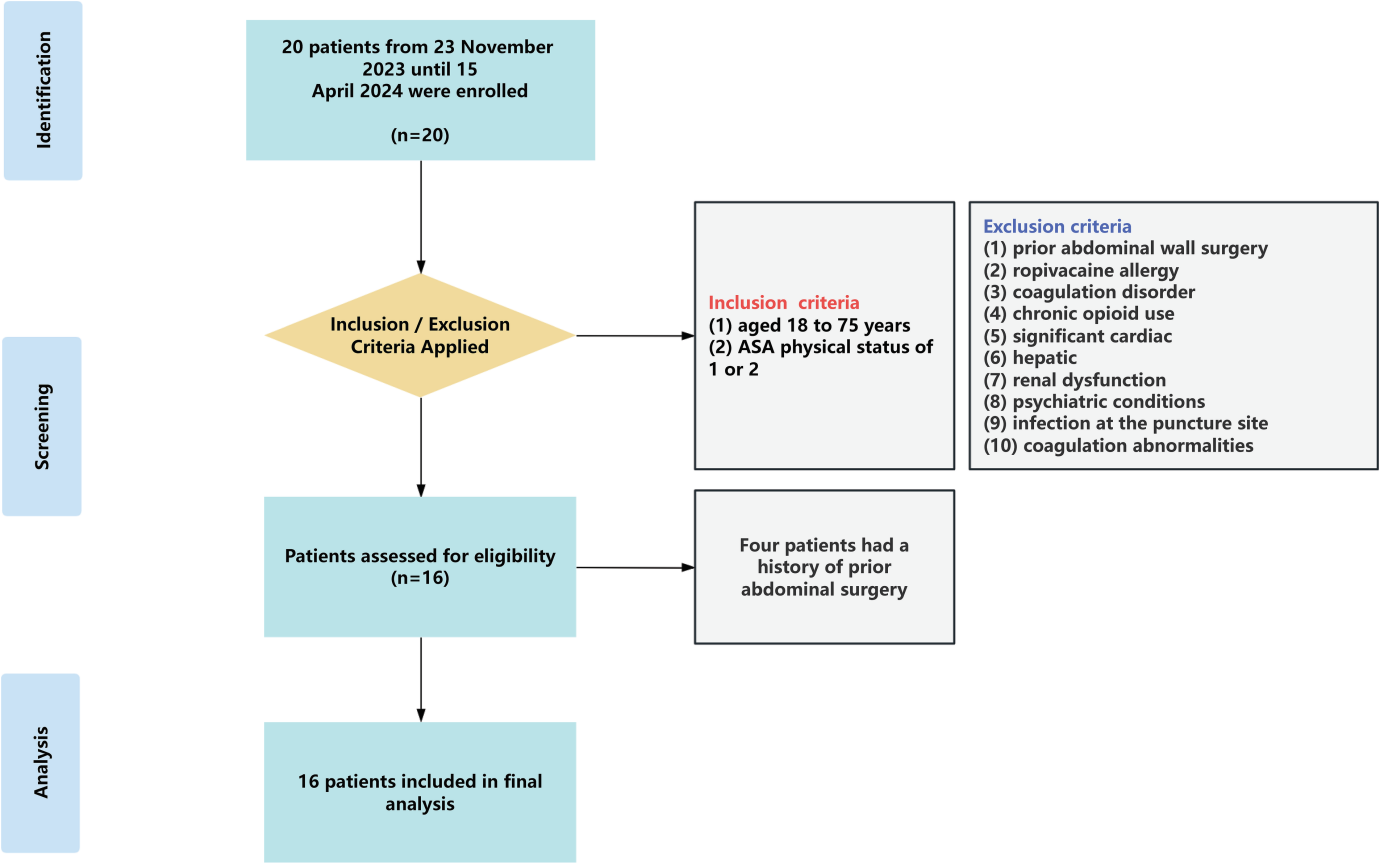
Patient inclusion flowchart. ASA: American Society of Anesthesiologists physical status classification.

A total of 20 patients were enrolled in this study. After excluding four patients with a prior history of abdominal surgery, data from 16 patients were included in the final analysis (Figure 3). Sixteen patients, including six females (38%), scheduled for elective LC were enrolled in the study. All participants underwent the bilateral novel approach US-TAP block and had CSBA assessed. Baseline characteristics included: median age of 42 years (range: 34–69); height, 168 cm (range: 158–181); weight, 71 kg (range: 57–93); and BMI, 25 kg/m^2^ (range: 20–29) (Table 1).

**Table 1:** Clinical characteristics.

Characteristics	*N* = 16
Age (yrs)	42 (34–69)
Sex	
Male	10 (62.5)
Female	6 (37.5)
ASA physical status: no. (%)	
1	7 (43.8)
2	9 (56.2)
Height (cm)	168 (158–181)
Weight (kg)	71 (57–93)
BMI (kg/m2)	25 (20–29)
Duration of Surgery (min)	69 (55–90)

Values are presented as median (range) or *n* (%). ASA: American Society of Anesthesiologists physical status classification.

### 3.2 Main results

Forty-five minutes after the novel bilateral US-TAP block, the cutaneous sensory block area (CSBA) was evaluated using a cold stimulus (alcohol-soaked gauze), as shown in Figures 2B–D. All applications of the novel US-TAP approach produced a cutaneous sensory effect. The median CSBA was 332 cm^2^ (IQR: 297–413 cm^2^, range: 258–466 cm^2^). The CSBA demonstrated a periumbilical distribution in all patients. Considering the bilateral US-TAP technique as two independent unilateral blocks, the following pattern was observed: 32 unilateral blocks were assessable 45 min post-block. The CSBA was most reproducible at mid-thoracic dermatomes. Along the anterior abdominal wall (midline to mid-clavicular lines), T10–T11 were universally anesthetized (32/32, 100%), with high extension to T9 (30/32, 93.8%) and T12 (26/32, 81.3%); cephalad spread to T8 occurred in 11/32 (34.3%), while no spread reached T6–T7 (0/32). Caudal involvement of L1 was observed in 7/32 (21.9%). Laterally (mid-clavicular to mid-axillary lines), coverage remained high at T9 (30/32, 93.8%) and T10 (31/32, 96.9%) but declined at T11 (28/32, 87.6%), T12 (16/32, 50.0%), and L1 (5/32, 15.6%); cephalad spread to T8 was infrequent (5/32, 15.6%), and absent at T6–T7. Overall, the distribution demonstrates a reliable band of anesthesia centered at T9–T11 with tapering cephalad and caudad spread, which was less extensive laterally than anteriorly (Table 2).

**Table 2:** Frequency of dermatomes blocked at 45 min after completion of bilateral novel ultrasound-guided transversus abdominis plane block approach in all 32 unilateral blocks.

Level	Anterior abdominal wall (*n* = 32)^*^	Anterolateral abdominal wall (*n* = 32)^&^
T6	0 (0)	0 (0)
T7	0 (0)	0 (0)
T8	11 (34.3)	5 (15.6)
T9	30 (93.8)	30 (93.8)
T10	32 (100)	31 (96.9)
T11	32 (100)	28 (87.6)
T12	26 (81.3)	16 (50)
L1	7 (21.9)	5 (15.6)

Values are presented as *n* (%). ^*^ The anterior abdominal wall denotes the region between the midline and the bilateral midclavicular lines; ^&^ The anterolateral abdominal wall denotes the region from the midclavicular lines to the midaxillary lines on both sides.

### 3.3 Secondary results

Among 16 patients, postoperative pain remained low at rest over the first 24 h (Table 3). Median resting NRS (0–10) increased from 1 (IQR, 1–2 [range, 0–3]) at 1 h to 3 (2–4 [2–4]) at 12–18 h, then declined to 2 (2–3 [1–4]) at 24 h. With coughing, pain scores were consistently higher than at rest, rising from a median of 2 (2–3 [1–3]) at 1 h to 3 by 12 h and remaining 3 through 24 h (IQR generally 2–4; maximum 5). Fourteen patients (88%) had resting NRS scores of 3 or lower within 24 h postoperatively, while 13 patients (81%) had NRS scores below 5 during coughing in the same period. No nerve block–related complications were observed in any patient.

**Table 3:** Numeric rating scale postoperatively.

NRS (*n* = 16)	1 h	4 h	12 h	18 h	24 h
Resting	1 (1–2 [0–3])	2 (2–4 [1–4])	3 (2–4 [2–4])	3 (2–4 [2–4])	2 (2–3 [1–4])
Coughing	2 (2–3 [1–3])	2 (3–4 [2–4])	3 (3–4 [2–5])	3 (3–4 [2–5])	3 (2–4 [1–5])

Values are median (IQR [range]). NRS, numeric rating scale; IQR, interquartile range.

## 4 Discussion

This study demonstrated that the novel US-TAP block yielded a broad dermatomal CSBA with less variability compared to subcostal (8) and posterior (9, 10) US-TAP approaches.

**Figure 4:**
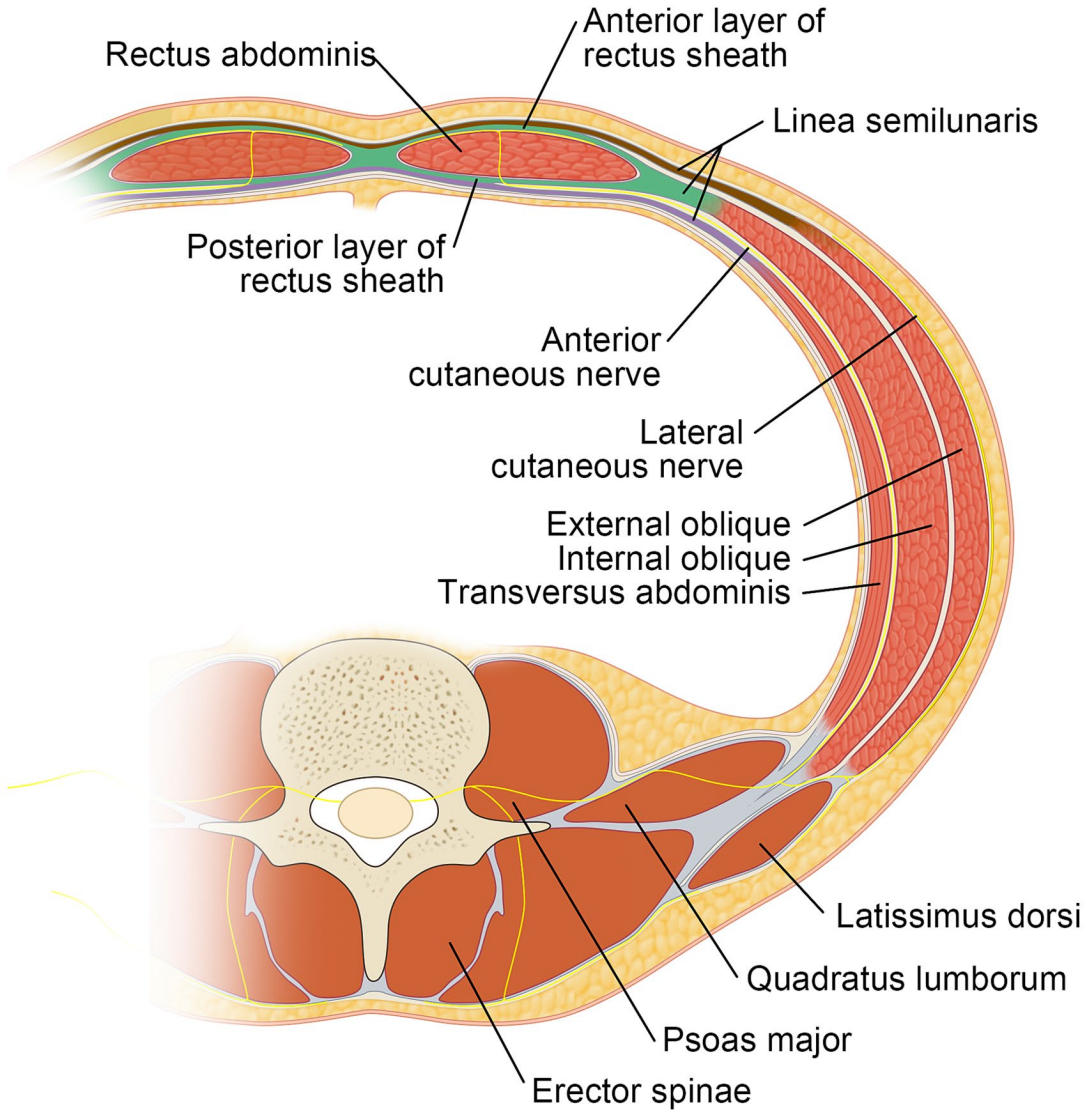
Cross-sectional view of the lower abdominal wall (at the T11 level) illustrating the path taken by a thoracolumbar nerve.

The novel US-TAP block is grounded in a more comprehensive understanding of the anatomical alignment between the intercostal, subcostal, and lumbar nerves. Upon exiting their respective intervertebral foramina, spinal nerves split into anterior and posterior rami. The anterior ramus subsequently branches into two primary nerves: the anterior and lateral cutaneous nerves. The anterior cutaneous branches (T6–T11) form the intercostal nerves, and the subcostal nerve (T12), along with the anterior branch of L1, innervate the skin and muscles of the anterior abdominal wall (12). These nerves are also targeted by the classical TAP block. However, the lateral cutaneous nerve, emerging from the anterior rami near the rib angle or midaxillary line, is often neglected (Figure 4). This anatomical variation may contribute to the substantial variability in the CSBA observed across different TAP block approaches. In this study, we employed a “one-stitch” technique to block the lateral cutaneous nerve, revealing that in 50% of unilateral CSBAs, the block extended to the lateral aspect of the anterior superior iliac spine (ASIS). This contrasts with prior research (3, 8, 13), which found that most blocks did not extend laterally to the ASIS. The findings of this study are promising, and the clinical insights gained are crucial for redefining TAP approaches, ensuring analgesic effects, and broadening its clinical application.

In contrast to the conventional subcostal US-TAP, the ultrasound probe placement for the novel US-TAP block in this study was positioned more laterally than the subcostal approach but more medially than the lateral approach. The T6–T8 intercostal nerves first course between the innermost and internal intercostal muscles, then enter the transversus abdominis plane at the costal margin. However, as noted by Barrington et al. (14), the anterior branches of the T6–T8 intercostal nerves may frequently pass directly into the rectus abdominis muscle near the costal margin, which could limit the effectiveness of a local anesthetic injection between the transversus abdominis and internal oblique muscles. This anatomical feature may contribute to incomplete blockade of these branches. In our study, we observed that the branches of T9–T11 were consistently affected by the block, whereas the T8, T12 and L1 branches were sometimes spared, which further highlights the anatomical variability in achieving complete blockade of the anterior abdominal wall. These factors should be considered when evaluating the overall efficacy of the novel US-TAP block. The T9–T11 intercostal nerves and the T12 subcostal nerve penetrate the transversus abdominis plane posterior to the midaxillary line (15). We hypothesize that the classical subcostal US-TAP may not reach T9–T11 because of its medial placement, while the lateral approach may miss T6–T8 due to its lateral position. Thus, we selected a more central placement to facilitate broad cephalad diffusion of local anesthetics, although further studies are required to validate this approach.

Our findings demonstrate a periumbilical distribution centered at T9–T11, corresponding to procedures where nociceptive input arises around the umbilicus, including three-port laparoscopic cholecystectomy, umbilical or paraumbilical hernia repair, and single-incision laparoscopic approaches. In contrast, when pain is localized to the upper abdomen (T8 or above), a paravertebral block generally offers more precise coverage, whereas lower abdominal pain with L1 involvement (such as Pfannenstiel incisions) may be better addressed by posterior or lateral TAP, or by iliohypogastric and ilioinguinal blocks. Therefore, this novel technique should be regarded as complementary rather than universally superior, especially in scenarios where periumbilical coverage is critical and wide lateral spread is unnecessary.

In our study, the CSBA following the novel US-TAP block predominantly encompassed a broad periumbilical region. Støving et al. (10) found that, after the posterior US-TAP, the CSBA primarily had a lateral distribution, with only a small portion (median area of 321 cm^2^) covering the medial infraumbilical region. Similarly, Salmonsen et al. (8) reported that the CSBA (median area of 174 cm^2^) following the subcostal US-TAP mostly covered the upper medial abdomen. In contrast, our study found that the CSBA (median area of 332 cm^2^) from the novel US-TAP approach covered a substantial portion of the abdomen centered around the umbilicus and extended to the inguinal region. However, the T12 and L1 nerves were not always consistently involved. T12 nerve coverage was observed in some patients, but not universally, and L1 nerve involvement was only noted in a few cases. This variability in nerve coverage could be due to differences in anatomy, the specific approach used, and the distribution of local anesthetic. To our knowledge, the median CSBA value measured in this study represents the largest reported to date. Notably, both Salmonsen et al. (8) and Chen et al. (16) used a subcostal approach to assess the CSBA, yet their findings varied significantly. These differences may be due to variations in target populations, measurement timing, and methodologies.

The broad CSBA associated with the novel US-TAP approach could guarantee analgesic effectiveness. The muscle-relaxing properties or systemic effects of the local anesthetic could significantly contribute to TAP-induced pain relief. However, several studies assessing the impact of TAP blocks on post-laparoscopic cholecystectomy pain have produced inconsistent results (17–20). This study showed that the majority of patients receiving the novel US-TAP approach reported mild postoperative pain. These findings may provide updated clinical evidence supporting the inclusion of the novel US-TAP approach in multimodal analgesic regimens for LC.

This study has several limitations. First, being a single-arm observational study, we could not establish a control group to assess CSBA differences in a randomized, blinded manner. Second, this study lacks power calculation, and may therefore have been underpowered to detect differences in outcome measures. The present study should therefore be characterized as a pilot study. Third, we assessed block effectiveness using a cold stimulus instead of postoperative pain relief. Cold and pain perceptions involve distinct sensory pathways, including different receptors, conduction mechanisms, and central integration (10). However, our goal was to achieve a straightforward and visual representation of the CSBA. Although the pinprick test may yield slight variations compared to the cold sensation test, visual documentation remained the primary focus of this study. Fourth, the multimodal regimen, including rescue analgesia, employed in this trial represents our institution’s long-standing standard for post-laparoscopic cholecystectomy pain control; however, its external generalisability is uncertain, and the observed analgesic efficacy may not translate to other centres or patient populations.

The novel US-TAP block may further contribute to the ongoing debate regarding the analgesic effectiveness and clinical relevance of TAP. We believe that a deeper understanding of TAP block effects, grounded in anatomical insights, is crucial. This study contributes updated knowledge on the clinical use of TAP block by mapping sensory distribution following the modified US-TAP technique. Randomized trials remain necessary to establish the clinical significance of the novel US-TAP approach.

In conclusion, the novel US-TAP approach produces a broad dermatomal CSBA, covering much of the abdominal wall around the umbilicus.

## Data Availability

The original contributions presented in the study are included in the article/Supplementary material, further inquiries can be directed to the corresponding authors.
